# Differences in the Pattern of Hemodynamic Response to Self-Face and Stranger-Face Images in Adolescents with Anorexia Nervosa: A Near-Infrared Spectroscopic Study

**DOI:** 10.1371/journal.pone.0132050

**Published:** 2015-07-07

**Authors:** Takeshi Inoue, Yuiko Sakuta, Keiichi Shimamura, Hiroko Ichikawa, Megumi Kobayashi, Ryoko Otani, Masami K. Yamaguchi, So Kanazawa, Ryusuke Kakigi, Ryoichi Sakuta

**Affiliations:** 1 Department of pediatrics, center for child development and psychosomatic, Dokkyo medical university Koshigaya hospital, Koshigaya, Saitama, Japan; 2 Faculty of Human Life Sciences, Jissen Women’s University, Hino, Tokyo, Japan; 3 Faculty of Science and Technology, Tokyo University of Science, Noda, Chiba, Japan; 4 Department of Integrative Physiology, National Institute for Physiological Sciences, Okazaki, Aichi, Japan; 5 Department of Psychology, Chuo University, Hachioji, Tokyo, Japan; 6 Department of Psychology, Japan Women's University, Kawasaki, Kanagawa, Japan; University of Toyama, JAPAN

## Abstract

There have been no reports concerning the self-face perception in patients with anorexia nervosa (AN). The purpose of this study was to compare the neuronal correlates of viewing self-face images (i.e. images of familiar face) and stranger-face images (i.e. images of an unfamiliar face) in female adolescents with and without AN. We used near-infrared spectroscopy (NIRS) to measure hemodynamic responses while the participants viewed full-color photographs of self-face and stranger-face. Fifteen females with AN (mean age, 13.8 years) and 15 age- and intelligence quotient (IQ)-matched female controls without AN (mean age, 13.1 years) participated in the study. The responses to photographs were compared with the baseline activation (response to white uniform blank). In the AN group, the concentration of oxygenated hemoglobin (oxy-Hb) significantly increased in the right temporal area during the presentation of both the self-face and stranger-face images compared with the baseline level. In contrast, in the control group, the concentration of oxy-Hb significantly increased in the right temporal area only during the presentation of the self-face image. To our knowledge the present study is the first report to assess brain activities during self-face and stranger-face perception among female adolescents with AN. There were different patterns of brain activation in response to the sight of the self-face and stranger-face images in female adolescents with AN and controls.

## Introduction

Eating disorders (EDs) are serious psychiatric disorders that predominantly affect female adolescents. In addition, EDs can cause serious medical and psychosocial morbidities. The prevalence rates for different types of EDs range from 0.3% to 1% in young females [1]. Anorexia nervosa (AN) is an ED characterized by distorted body image, self-induced starvation, and excessive weight loss with a pathological fear of becoming fat. Psychological, social, genetic, and cognitive deficits play a role in the onset and the maintenance of EDs.

Body image distortion is a major symptom of AN. It is widely known that AN patients perceive their own body, particularly body parts (e.g. thighs, abdomen or face) to be fatter than they are.[2]. Neural basis of body image distortion has gradually become clear in recent years. Over the past decade, several functional neuroimaging studies have researched the possible neurofunctional correlates of body images distortion in patients with AN and other EDs. These studies have documented different neural activation patterns between patients and controls [3–6].

The face is an important component of self-concept, and it is the least occluded part of the body that plays crucial role in interpersonal communications. AN patients see and feel their own face as fatter than it actually is. However, few studies have attempted to investigate the self-face perception in patients with AN. Facial perception is related to the part of the temporal cortex [7–9] as well as body perception [10, 11]. Research on self-face perception has been conducted for more than a decade and several studies have suggested that self-face perception involves distinct brain mechanisms compared with stranger-face perception [12–15]. Therefore, we hypothesized that subjects with AN who present with own-body image distortion have different self-face perception compared with controls. To our knowledge, there are no published reports on the perception of self-face and stranger-face in patients with AN. Thus, the purpose of this study was to compare neuronal correlates of self-face and stranger-face (female) perception in female adolescents with AN and without AN (controls).

Near infrared spectroscopy (NIRS) is a noninvasive neuroimaging methodology that monitors blood volume and oxygenation in the brain [16, 17]. The general rationale of NIRS is that it provides an index of brain function by assessing changes in the concentration of oxygenated hemoglobin (oxy-Hb), deoxygenated hemoglobin (deoxy-Hb), and total-hemoglobin (total-Hb) using measurements of the diffuse transmittance of near-infrared light at an appropriate range of wavelengths. It is widely accepted that brain activity is related to regional changes in blood flow and oxygenation [18]. NIRS has been used for more than a decade to examine brain activity during various cognitive tasks [19–23]. With regard to facial perception, NIRS has been used to measure neural activities in the temporal area. Several lines of evidence have suggested that the hemodynamic response in the temporal cortex of the brain is activated during face processing [24–31].

Compared with other neuroimaging techniques such as functional magnetic resonance imaging (fMRI), NIRS is completely silent, providing a non-intrusive environment for the patients. Furthermore, NIRS can be conducted in a brief period of time, and it requires less restriction of the body and the head. NIRS has been utilized for the assessment of brain activities in children with psychiatric disorders including AN, while the subjects performed cognitive tasks. Nagamitsu et al. reported that the analysis of information obtained by NIRS revealed that the prefrontal hemodynamic response during a cognitive task (word fluency task) differed between patients with AN and controls [32]. In the current study, we used NIRS to measure the hemodynamic responses in patients with AN and in age- intelligence quotient (IQ)-matched controls while they viewed self-face and stranger-face photographs in full color.

The purpose of this study was to compare the neuronal correlates of viewing self-face and stranger-face images in female adolescents with AN and in controls using a NIRS system which was placed on the bilateral temporal regions. There were different patterns of brain activation in response to the sight of the self-face and stranger-face in female adolescents with AN and controls.

## Methods

### 2.1 Participants

The current study involved 15 female adolescents who fulfilled the criteria for AN in the 4th text revision edition of the Diagnostic and Statistical Manual of Mental Disorders (DSM-IV-TR). The AN subtype of all patients was restrictive. None of the patients had other psychiatric or neurological disorders including past history of pervasive developmental disorder. The Children's version of the Eating Attitude Test-26 (ChEAT-26), a screening instrument which is widely used to measure the characteristics of EDs in children [33] was administered to all participants. ChEAT-26 has been commonly used as an index of the symptoms of AN; a high score indicates a tendency toward EDs. The patients were recruited from the Department of Pediatrics, Center for Child Development and Psychosomatic Medicine, Dokkyo Medical University Koshigaya Hospital in Japan. They were tested either 2–4 weeks after admission or at the first visit to the clinic. All female adolescents with AN were in the acute phase, yet their general conditions were stable enough to walk and have conversation with others. Their mean age and body mass index (BMI) at the time of study were 13.8 years [standard deviation (SD) = 1.59] and 14.4 kg/m^2^ (SD = 2.20), respectively (Table 1). Their severity of weight loss was clinically severe. The control group comprised 15 female volunteers, who were recruited from a local school. They had no history of psychiatric or neurological disorders, and no irregularities in eating behavior. Their mean age and BMI at the time of study were 13.1 years (SD = 1.10) and 18.7 kg/m^2^ (SD = 2.15), respectively (Table 1). All the participants were right-handed. Written informed consent (as outlined in PLOS consent form) was obtained from the individuals in this manuscript. This study was approved by the Ethical Committee of the Dokkyo Medical University Koshigaya Hospital (hosp-k 24016).

**Table 1:** Clinical characteristics of all participants.

	AN (n = 15)	Control (n = 15)	*P*
Age (years)	13.8±1.59	13.1±1.10	0.0671
BMI (kg/m2)	14.4±2.20	18.7±2.15	<0.0001
BMI-SDS	-3.1+1.80	-0.25±0.71	<0.0001
ChEAT26	34.0±20.7	8.7±3.71	0.0008
IQ	102.3±13.8	104.3±9.32	0.4465

Data is presented as mean ± SD (Mann-Whitney *U* test)

### 2.2 Stimuli and design

**Fig 1:**
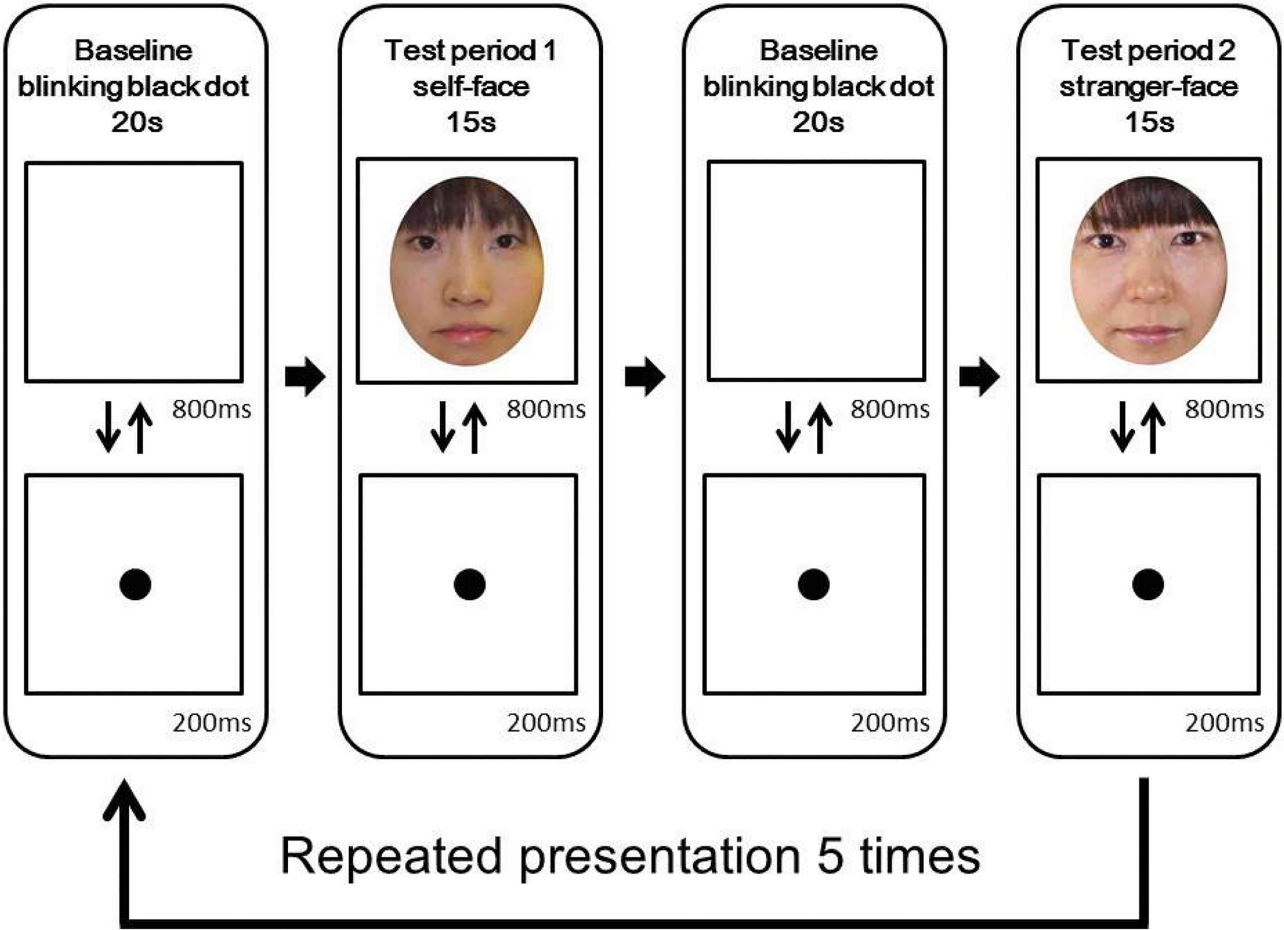
Experimental procedure. In each trial, the baseline phase consisted of visual stimuli presenting images of a black dot, and its duration was fixed at 20s. The test phases consisted of self-face and stranger-face visual stimuli. The duration of each test period was 15s. The presentation order of test phases 1 and 2 was alternated for each participant.

The stimuli presentation sequence consisted of baseline and test periods. For the test period, there were two stimulus conditions: self-face and stranger-face conditions. In those conditions the participants viewed self-face or stranger-face color photographs, respectively (Fig 1). The order of the conditions was counterbalanced across the participants. The self-face photograph was the participant’s own face. The stranger-face photograph was the face of another age-matched female adolescent with AN in the case of the AN group and another age-matched control in the case of the control group. To eliminate the external features of the faces, we removed the hair from the face images so that only the internal features were visible. The sizes of the stimuli were approximately 13 × 10 deg for the faces and 3.5 × 3.5 deg for the blinking black dots.

The duration of the test period was fixed at 15s. Each face image was shown 15 times in one test period. The duration of each face image was 800ms, and the 200ms inter-stimulus interval was filled by the presentation of a fixation point (a blinking black dot). Each test period was preceded by a baseline period that was fixed at 20 s. During the baseline period, the screen of the monitor was filled with a white uniform blank. The blank was presented for 800 ms, and a 200 ms inter-blank interval was filled with the same fixation point (a blinking black dot) used in the test period. The results obtained from viewing the uniform blank were used as the baseline.

### 2.3 Procedure

Each participant was tested while sitting in a chair and facing a computer screen approximately 50 cm away. The participants passively watched the stimuli while their brain activity was measured, and they were allowed to watch the stimuli for as long as they were willing. The behavior of the subjects was observed by three evaluators, and the viewing time was recorded using a video system that allowed tracking the line of sight during the experiment.

### 2.4 Recording

Real time cerebral-cortex imaging and measurement were conducted using the Hitachi ETG-4000 and ETG-7100 systems (Hitachi Medical, Chiba, Japan). The NIRS wavelengths used for measurement were the same for both systems (695 and 830 nm); therefore, we believe that the differences in the specifications of the two systems had no effect on our results. We recorded 24 channels simultaneously, with 12 channels for the right temporal area and 12 for the left. Both instruments generate two wavelengths of NIR (695 nm and 830 nm) and measured the time courses of the levels of oxy-Hb, deoxy-Hb, and their sum (total-Hb) with 0.1 s time resolution.

The NIRS probes contained nine optical fibers (3 × 3 arrays). Of the nine fibers, five were emitters and four were detectors. The distance between the emitters and detectors was set at 3 cm. Each pair of adjacent emitting and detecting fibers defined a single measurement channel.

**Fig 2:**
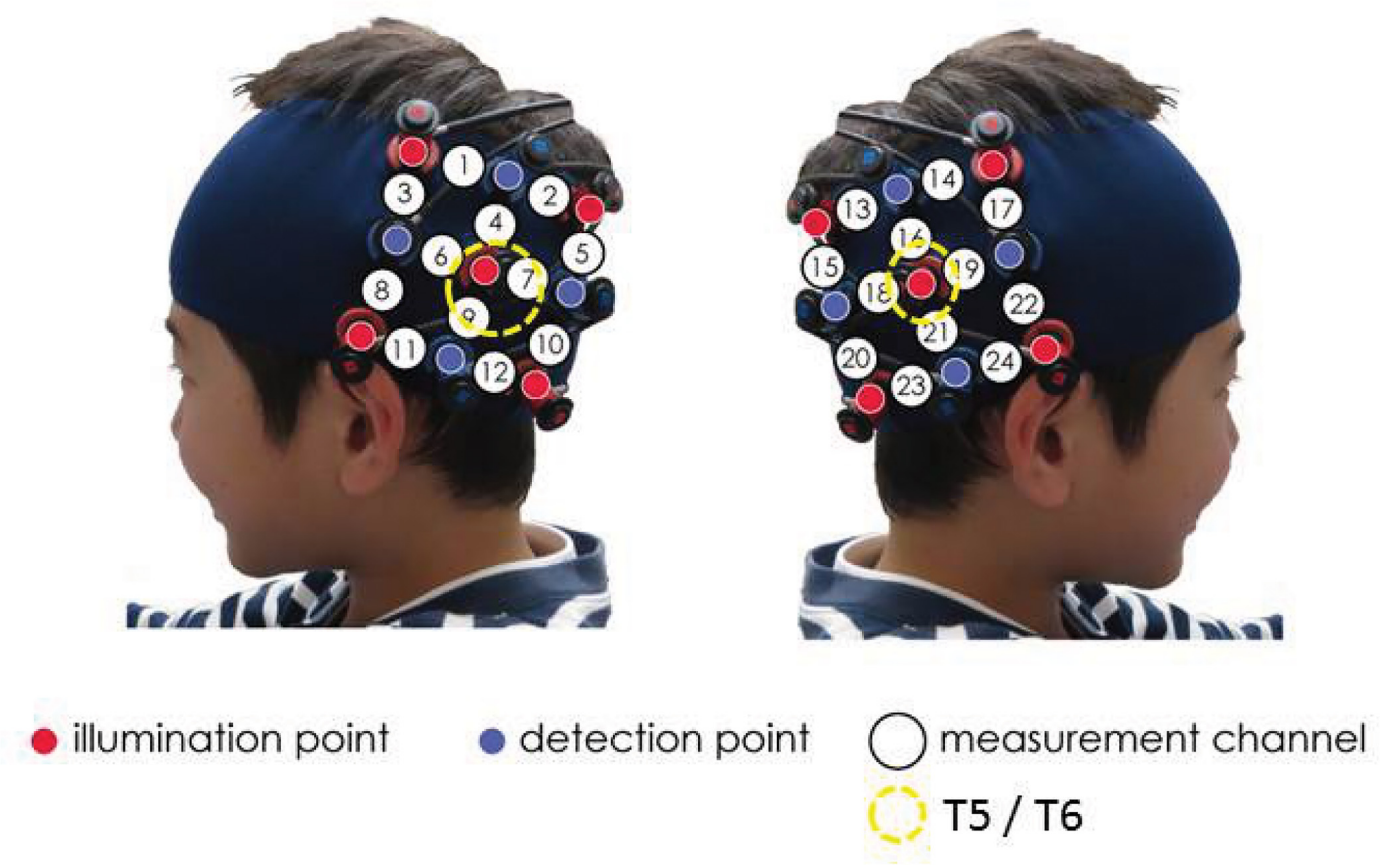
Placement of NIRS probes. The probes were placed on the left and right temporal areas centering at T5 and T6 of the International 10–20 system. T5/T6 is indicated by the yellow circle. The distance between the probes was set at 3 cm.

The placement of the participants’ probes was set on the bilateral temporal area centered at T5 and T6 according to the International 10–20 system [34] (Fig 2). This was the same location of probes used by Nakato et al. [24]. When the probes were positioned, the experimenter ensured that the fibers were attached to the participants’ scalp correctly. The Hitachi ETG-4000 and 7100 systems automatically detect whether the contact adequately measures emerging photons for each channel. Channels were rejected from the analysis if adequate contact between the fibers and participants’ scalp could not be achieved because of hair interference.

### 2.5. Data analysis

The NIRS data analysis was performed in a manner similar to that of Nakato et al. [24]. Before performing data analysis, we monitored the video recording of the participants’ behavior to evaluate adequate trials for the analysis. We removed the trial from the analysis when either of the following occurred: [1] accumulative looking time within the trial did not reach 5 s, or [2] movement artifacts were detected by the analysis of sharp changes in the time series of the raw NIRS data.

The raw data on oxy-Hb, deoxy-Hb, and total-Hb concentrations from each channel were digitally band-pass-filtered at 0.02–1.0 Hz to remove the noise caused by the heartbeat pulsations or any longitudinal signal drift. The mean concentration of each channel within a subject was then calculated by averaging data across the trials in a time series with a 0.1 s resolution.

Based on the mean concentrations in the time series and the “baseline” values (mean concentration at 3 s before the trial), we calculated the Z-scores for oxy-Hb, deoxy-Hb, and total-Hb in the self-face and stranger-face conditions for each channel within a subject [24–30, 35]. The Z-score at each time point was used to indicate the deviation of hemodynamic response from the “baseline” during the presentation of facial images. The Z-scores were calculated separately for oxy-Hb, deoxy-Hb, and total-Hb in the self-face and stranger-face conditions. A previous NIRS study reported that the inferior region in the temporal cortex is activated more than the other regions in the temporal cortex when participants looked at facial images [24]. Therefore, the Z-scores obtained from the set of 6 channels within the left inferior temporal area and the set of 6 channels in the right inferior temporal area were each averaged across trials for every participant, as previously described [24]. Although the raw NIRS data were relative values and could not be averaged directly across participants or channels, the normalized data (i.e., Z-scores) could be averaged regardless of the unit [17, 36]. The Z-score is a reliable indicator of the concentration changes because the analysis is independent of the differential path length factor.

**Fig 3:**
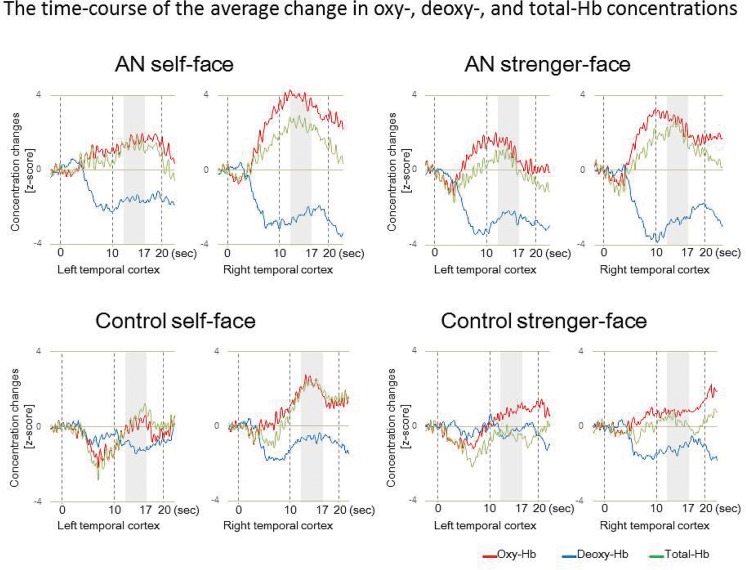
The time-course of the average change in oxy-Hb, deoxy-Hb, and total-Hb concentrations. The time-course of the average change in oxy-Hb, deoxy-Hb, and total-Hb concentrations in the temporal regions of the participants during the presentation of the self-face and stranger-face conditions. Zero on the horizontal axis represents the onset of the test period and the duration of the test period was fixed at 15 s. The area in gray represents the zone that was averaged for statistical analysis.

Consistent with our previous studies using NIRS [24, 27–29], the hemodynamic response of oxy-Hb increased gradually during the test period and reached a plateau approximately 12 s after the stimuli onset (Fig 3). Thus, we used the mean Z-score for the 12–17 s interval of the test period for the statistical analysis. To examine the possibility that the activity resulting from the self-face or stranger-face stimuli were greater or smaller than 0, we conducted two-tailed one-sample t-tests with the mean Z-scores for oxy-Hb deoxy-Hb, and total-Hb concentrations in each condition. The statistical significance threshold was set to P < 0.05.

## Results

### 3.1. Clinical characteristics of participants

The characteristics of the study participants are listed in Table 1. There was no significant difference in age or IQ between the two groups. The BMI and BMI-standard deviation score (BMI-SDS) values in the AN group were significantly lower compared with those in the control group (*P* < 0.0001, Mann-Whitney *U*-test), and the ChEAT-26 [33] scores in the AN group were significantly higher compared with those in the control group (*P* = 0.0008, Mann-Whitney *U*-test).

### 3.2. Available trials for statistical analysis

Data for 30 participants who looked at faces in five trials in both the self-face and stranger-face conditions were obtained. The date were averaged across trials and across participants for each condition (self-face: mean = 4.87 trials, SD = 0.51; stranger-face: mean = 4.93 trials, SD = 0.59). There was no significant difference between the numbers of available trials under each condition.

### 3.3. NIRS measurements

**Fig 4:**
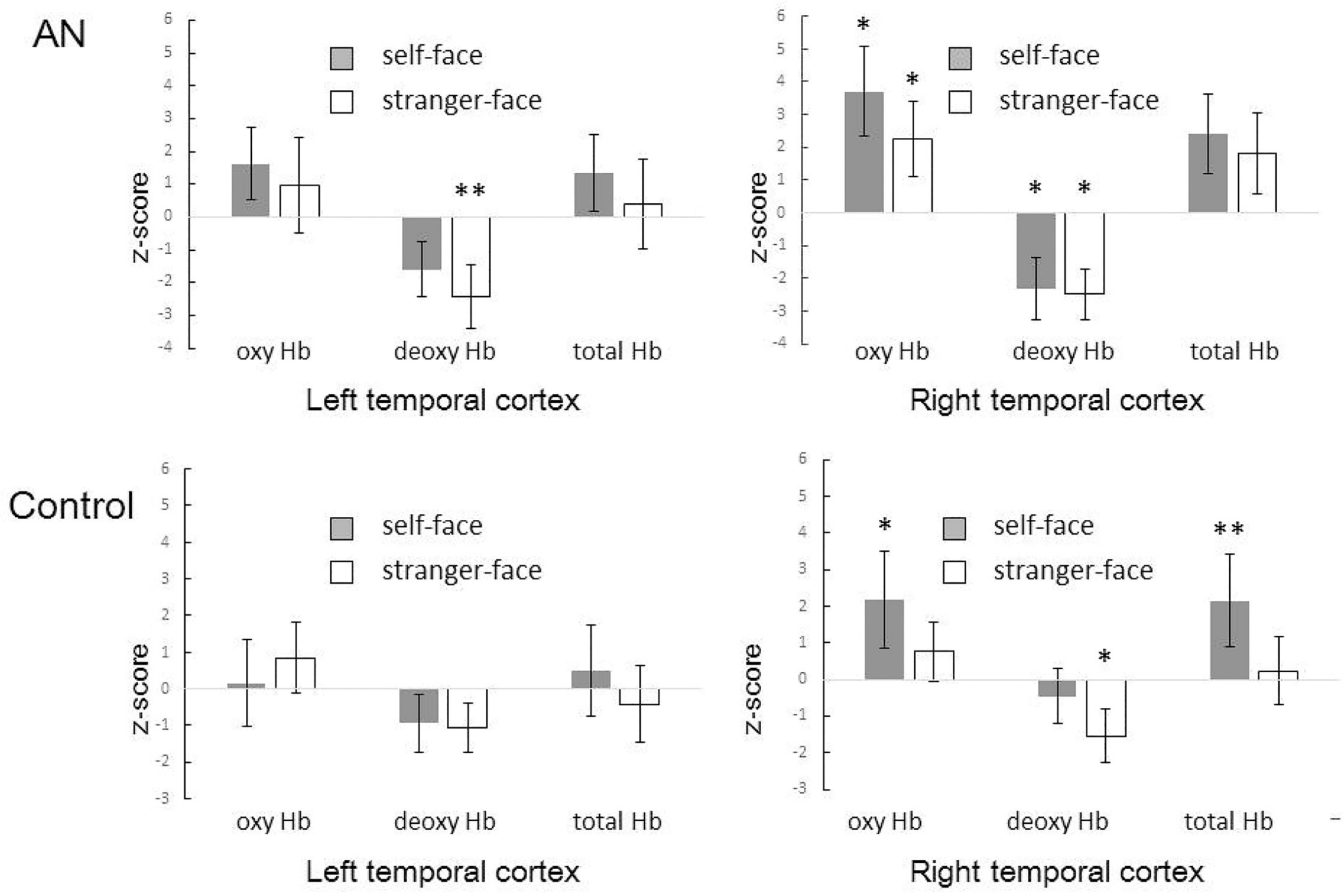
The mean Z-score during the 12–17 s period. The mean Z-score during the 12–17 s period after the onset of stimulation in the left and right temporal cortices. The gray bars represent the self-face condition and the white bars represent the stranger-face condition. The error bars represent one SD. The left and right panels indicate the data for the left and right temporal cortices, respectively; the top and bottom panels indicate the data from the AN and control groups, respectively. The statistical significance threshold was set to *P < 0.05.

At the wavelengths measured by the Hitachi ETG-4000 and ETG-7100 systems (695 and 830 nm), the estimations of oxy-Hb and total-Hb concentrations are more sensitive than those of deoxy-Hb concentrations [22, 37]. However, when compared with deoxy-Hb and total-Hb concentrations, the oxy-Hb concentration is a better indicator of changes in regional cerebral blood flow [38]. The time-course of the average change in oxy-Hb, deoxy-Hb, and total-Hb concentrations was measured in the participants during the presentation of the self-face and stranger-face images (Fig 3), and Z scores were calculated based on the differences between the values after stimulus onset and the background values (Fig 4).

The Z-scores for oxy-Hb concentrations in the right temporal cortices of the AN group were significantly greater than 0 in both the self-face and stranger-face conditions (*P* = 0.015 and *P* = 0.032, respectively). In contrast, the Z-scores for oxy-Hb concentrations in the right temporal cortices of the control group were significantly greater than 0 in the self-face condition (*P* = 0.011), but not in the stranger-face condition (*P* = 0.35).

The Z-scores for deoxy-Hb concentrations in the right temporal cortices of the AN group were significantly less than 0 in both of the self-face and stranger-face conditions (*P* = 0.039 and *P* = 0.011, respectively), and the Z-scores for deoxy-Hb concentrations in the right temporal cortices of the control group were significantly less than 0 in the stranger-face condition (*P* = 0.012), but not in the self-face condition (*P* = 0.38).

The Z-scores for total-Hb concentrations in the right temporal cortices of the AN group were not significantly different from 0 in either condition. In contrast, the Z-scores for total-Hb concentrations in the right temporal cortices of the control group were significantly higher than 0 in the self-face condition (*P* = 0.0068).

None of the Z scores (oxy-Hb, deoxy-Hb, or total-Hb) were significantly different from 0 in the left temporal cortices with the exception of the Z-scores for deoxy-Hb concentrations, which were significantly less than 0 in the AN group (*P* = 0.0015).

## Discussion

In this study, we hypothesized that patients with AN who present with own-body image distortion have different self-face perception compared with controls. To compare the neuronal correlates of viewing self-face images and stranger-face images between AN and controls, we measured hemodynamic responses while the participants viewed full-color self-face and stranger-face photographs using NIRS. NIRS is a noninvasive neuroimaging method that does not require physical restraint of the subject and has a brief examination time compared with other neuroimaging methods. Thus, it is suitable for children and patients in the acute phase of AN. The objective of this study was to use NIRS to investigate the brain activity of participants in the AN and control groups while they viewed full-color self- and stranger-face photographs.

The fusiform gyrus and superior temporal sulcus, which are located in the inferior temporal lobe play important roles in face recognition [7, 8]. Therefore, the placement of the NIRS’ probes was attached to the bilateral temporal regions of the participants, as in Nakato et al. [24]. The Z-scores obtained from the six channels within the inferior temporal areas were analyzed.

NIRS monitors blood volume and oxygenation in the brain. When deoxy-Hb and total-Hb concentrations are compared, the oxy-Hb concentration reflects changes in regional cerebral blood flow more accurately [38]. In our study, the hemodynamic response, as indicated by oxy-Hb, increased gradually during the test period (i.e., photograph-viewing period) and was found to occur predominantly in the right hemisphere, suggesting that the brain activity of facial perception mainly occur on the right side. A recent neuroimaging study also reported that the main aspects of facial processing occur predominantly in the right hemisphere [9]. Sugiura et al. reported that activation selective to the self-face compared with the stranger-face was observed in the right occipito-temporo-parietal junction [12]. Furthermore, in recent surveys that adopted a stringent statistical threshold, the neural response specific to the self-face was detected predominantly in the right hemisphere [13, 14]. Here, the AN and control groups comprising adolescent females differed in their changes in brain activity in the right temporal cortices in response to viewing self-face and stranger-face photographs. In other words, the oxy-Hb concentrations of the control group significantly increased only in the self-face condition, whereas oxy-Hb concentrations of the AN group significantly increased in both conditions.

These findings suggest that self-face and stranger-face were normally distinguished in controls, which is in line with previous studies [12–15], whereas self-face and stranger-face were processed in the same manner in patients with AN. Body image distortion is a key symptom of AN, and it plays a role in the onset and maintenance of AN [2]. The face is one of the most important a part of the body, and body (including a face) recognition is related to a part of the temporal cortex [10, 11]. Additionally the fusiform gyrus [3–5] and temporal gyrus [39, 40], which play important roles in facial perception, are also related to self-body image perception in AN. The reason why patients with AN could not distinguish between self-face and stranger-face in this study may be related to their body image distortion.

Some studies have focused on the possible presence of cerebral functional disturbance in the development of AN. Cognitive deficits in body image, attention, learning, cognitive flexibility, memory, emotion, and facial affect have been observed in patients with AN by various types of neuropsychological examinations. However, it is still uncertain whether these cognitive deficits precede ED or appear as a consequence of malnutrition [41–43]. In the current study, all female adolescents with AN were in the acute phase. In the future, we intend to investigate brain activity again after recovery.

In conclusion, to our knowledge, the present study is the first to assess the brain activity during self-face and stranger-face processing in adolescents with AN. We have demonstrated that patterns of brain activation in response to the sight of the self-face and stranger-face are different between female adolescents with AN and controls. The face is an important part of the body; moreover, we found hemodynamic differences in the right temporal area, which corresponds to structures known to contribute to facial perception and self-body image perception [3–5, 39, 40]. Thus, different patterns of brain activation in response to the sight of the self-face and stranger-face in this study may be related to body image distortion in AN.
